# Elucidating the Mechanism of VVTT Infection Through Machine Learning and Transcriptome Analysis

**DOI:** 10.3390/ijms26031203

**Published:** 2025-01-30

**Authors:** Zhili Chen, Yongxin Jiang, Jiazhen Cui, Wannan Li, Weiwei Han, Gang Liu

**Affiliations:** 1 Academy of Military Medical Sciences, Beijing 100850, China; 2 Key Laboratory for Molecular Enzymology and Engineering of Ministry of Education, School of Life Sciences, Jilin University, 2699 Qianjin Street, Changchun 130012, China; weiweihan@jlu.edu.cn (W.H.)

**Keywords:** transcriptome, machine learning, gene expression, feature selection, vaccinia virus Tiantan strain

## Abstract

The vaccinia virus (VV) is extensively utilized as a vaccine vector in the treatment of various infectious diseases, cardiovascular diseases, immunodeficiencies, and cancers. The vaccinia virus Tiantan strain (VVTT) has been instrumental as an irreplaceable vaccine strain in the eradication of smallpox in China; however, it still presents significant adverse toxic effects. After the WHO recommended that routine smallpox vaccination be discontinued, the Chinese government stopped the national smallpox vaccination program in 1981. The outbreak of monkeypox in 2022 has focused people’s attention on the *Orthopoxvirus*. However, there are limited reports on the safety and toxic side effects of VVTT. In this study, we employed a combination of transcriptomic analysis and machine learning-based feature selection to identify key genes implicated in the VVTT infection process. We utilized four machine learning algorithms, including random forest (RF), minimum redundancy maximum relevance (MRMR), eXtreme Gradient Boosting (XGB), and least absolute shrinkage and selection operator cross-validation (LASSOCV), for feature selection. Among these, XGB was found to be the most effective and was used for further screening, resulting in an optimal model with an ROC curve of 0.98. Our analysis revealed the involvement of pathways such as spinocerebellar ataxia and the p53 signaling pathway. Additionally, we identified three critical targets during VVTT infection—ARC, JUNB, and EGR2—and further validated these targets using qPCR. Our research elucidates the mechanism by which VVTT infects cells, enhancing our understanding of the smallpox vaccine. This knowledge not only facilitates the development of new and more effective vaccines but also contributes to a deeper comprehension of viral pathogenesis. By advancing our understanding of the molecular mechanisms underlying VVTT infection, this study lays the foundation for the further development of VVTT. Such insights are crucial for strengthening global health security and ensuring a resilient response to future pandemics.

## 1. Introduction

The vaccinia virus (VV), which is used as a smallpox vaccine, belongs to the family *Poxviridae* and genus *Orthopoxvirus*. It is a complex double-stranded DNA virus that only replicates and forms special viral particles in the cytoplasm of the host [1]. The VV genome size is 185–200 kb, and it can encode about 200 different proteins [2]. The smallpox vaccine developed from VV has played a crucial role in the global smallpox eradication campaign [3]. Furthermore, research on VV as a vector has made significant advancements in various areas [4]. VV has proven valuable in investigating vaccine vectors and exogenous gene expression systems [5] and is extensively utilized in genetic engineering for these purposes [6,7]. Moreover, live genetically engineered vector vaccines utilizing VV as vectors have been successfully developed [8,9]. Presently, key areas of focus in VV vector research include reducing the side effects of VV vaccines, enhancing the efficacy of vector vaccines, and streamlining preparation protocols [10,11,12,13,14].

The largest outbreak of monkeypox in history began in May 2022 and has rapidly spread across the globe ever since [15]. To date, the most effective way to prevent or control an *Orthopoxvirus* outbreak is through vaccination [16]. Research on antibody responses to *Orthopoxvirus* species suggests perfect cross-immunity between smallpox and monkeypox [17]. Attack rates in individuals with and without vaccination scars indicated that smallpox vaccination (discontinued in 1980) imparted approximately 85% protection against monkeypox [18]. Data suggest that prior immunization with the smallpox vaccine may have a protective effect against the monkeypox virus and may improve clinical manifestations of infection [19,20,21]. However, numerous reports of adverse reactions following smallpox vaccination have been documented in the United States, some of which are even life-threatening [22]. Second-generation vaccines have contraindications. Third-generation vaccines, although safer for immunocompromised populations, require two doses, which is an impediment to rapid outbreak response and still has side effects [23,24]. The Bipartisan Commission on Biodefense pointed out that smallpox and other *Orthopoxviruses* pose significant threats to the United States and the world because of their potential for weaponization, accidental release, and vulnerability of populations who stopped routinely vaccinating against smallpox in the 1970s [25]. Therefore, further research is needed to prepare for the potential hazards they may bring.

With the advancement of omics technologies, studies utilizing genomics and proteomics to investigate VV have been published, enhancing our understanding of the molecular mechanisms of VV infection in cells [26,27]. Machine learning has demonstrated significant potential in handling complex and diverse modern biological data [28,29,30]. It effectively overcomes the limitations of traditional methods in dealing with high-dimensional data, capturing non-linear relationships, processing noise, and integrating multiple data types, thus better elucidating the complex patterns and biological mechanisms within [31]. This capability not only makes data analysis more precise and efficient but also provides deeper insights into research [32]. Feature selection is a crucial step in machine learning, especially when dealing with high-dimensional data [33]. The goal of feature selection is to identify a subset of features that contribute most significantly to the model’s predictive performance, thereby reducing model complexity, improving generalization ability, and lowering computational costs. In transcriptome analysis, feature selection can help identify the most relevant genes, further elucidating their roles in biological processes [34]. Thus, feature selection holds significant potential for effectively mining transcriptome data.

The vaccinia virus Tiantan strain (VVTT) plays an irreplaceable role as a vaccine strain for the eradication of smallpox in China [35]. After the WHO recommended that routine smallpox vaccination be discontinued, the Chinese government stopped the national smallpox vaccination program in 1981 [36]. The emergence of studies on VVTT-specific humoral immunity timing and cross-immunity with monkeypox only occurred following the outbreak of monkeypox in China [37,38,39,40]. Nonetheless, there are limited reports on the safety and toxic side effects of VVTT. To address future demands, we utilize transcriptome analysis and machine learning for feature selection to pinpoint significantly differentially expressed genes in 293A cells under VVTT treatment. This approach aims to deepen our understanding of VVTT’s impact on cells and explore the pathogenesis of smallpox vaccine side effects, offering theoretical insights for future vaccine development.

## 2. Results and Discussion

### 2.1. Differential Gene Analysis

**Figure 1:**
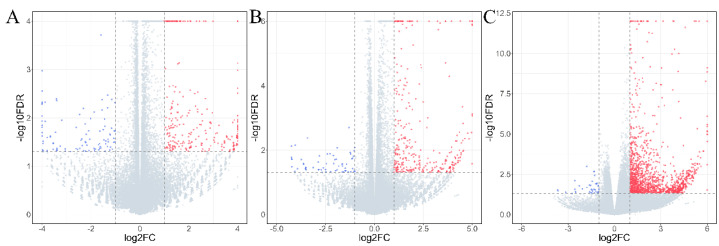
Volcano plots of differentially expressed genes. Results of differential gene analysis for mock vs. VVTT−6 h (**A**), mock vs. VVTT−12 h (**B**), and mock vs. VVTT−24 h (**C**) groups. Red indicates up-regulated genes; blue indicates downregulated genes.

The results of the differential gene analysis are shown in Figure (Figure 1). The data indicate that the number of differentially expressed genes (DEGs) increases with the duration of VVTT infection. Specifically, the numbers of DEGs at 6 h, 12 h, and 24 h post-infection were 360, 391, and 1449, respectively. Additionally, there was a general upward trend in gene expression levels as the infection time increased.

### 2.2. Protein–Protein Interaction Network

**Figure 2:**
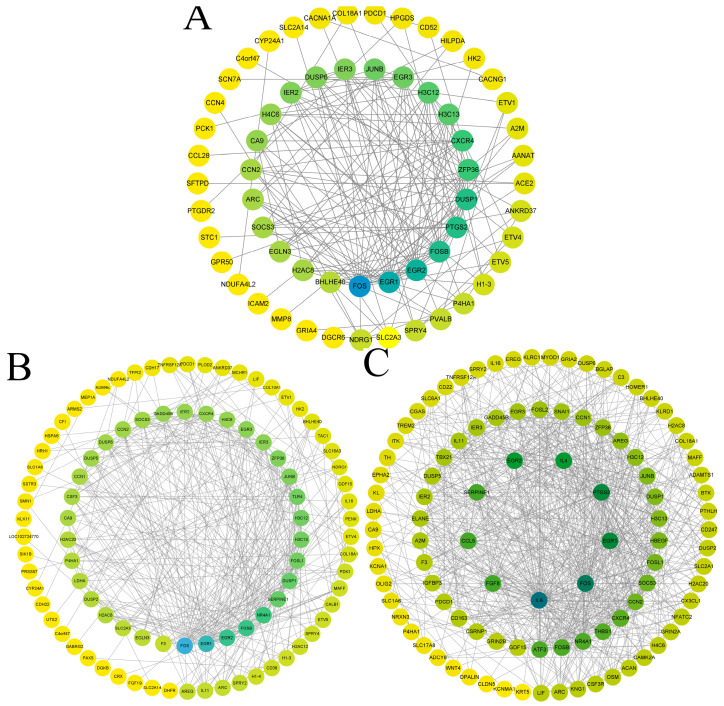
PPI networks for (**A**) mock vs. VVTT−6 h, (**B**) mock vs. VVTT−12 h, and (**C**) mock vs. VVTT−24 h groups. Darker colors indicate higher degree. Each node represents a corresponding protein and each edge represents the interaction between two proteins.

To identify potential key genes, we constructed a protein–protein interaction (PPI) network using the genes identified in the differential gene analysis. Figure 2 displays approximately the top 100 genes. Based on their degree, we selected the top 20 genes from each group (Table 1). Genes such as DUSP1, EGR1, EGR2, EGR3, FOS, FOSB, FOSL1, JUNB, and ZFP36 appear multiple times, suggesting their possible roles in VVTT-infected cells.

### 2.3. Enrichment Analysis

To further investigate the potential mechanisms of VVTT infection in cells, we performed GO and KEGG pathway analyses on the DEGs (Figures S1–S6, Supplementary Materials). The GO analysis results suggest that processes such as the cellular response to decreased oxygen levels, cellular response to hypoxia, cellular response to oxygen levels, negative regulation of cell adhesion, positive regulation of cell development, regulation of nervous system development, response to glucocorticoids, and response to hypoxia may play significant roles when cells are infected with VVTT. Additionally, the GO terms “response to decreased oxygen levels” and “response to oxygen levels” appeared in the results of all three analyses, suggesting that they may play significant roles.

Then, we selected the top 20 KEGG pathways from each group for display (Table 2). Combined with the targets identified in the PPI network analysis, pathways such as amphetamine addiction, circadian entrainment, MAPK signaling pathway, osteoclast differentiation, C-type lectin receptor signaling pathway, and TNF signaling pathway may be associated with VVTT infection in A293 cells. Further analysis of the genes enriched in these pathways indicated that genes such as ARC, FOS, EGR2, EGR3, IL6, JUN, and JUNB may warrant further attention.

### 2.4. Initial Gene Screening Based on Machine Learning

**Figure 3:**
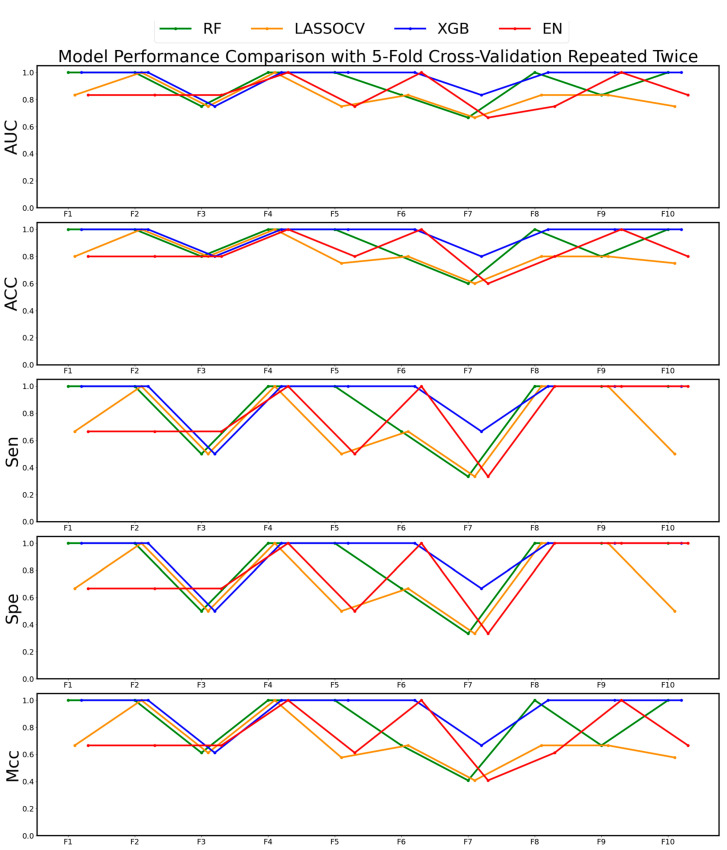
AUC, ACC, Mcc, Sen, and Spe of each algorithm in each fold of the five-fold cross-validation repeated twice.

To further investigate potential key genes, we used four machine learning algorithms—RF, LASSOCV, EN, and XGB—to preliminarily screen key genes from the expression matrix. We performed five-fold cross-validation twice, and the evaluation parameters obtained in each fold are shown in Figure 3. The comparison revealed that XGB produced the best results, so we focused subsequent analyses on further screening using XGB. Additionally, we combined the genes selected by the four algorithms with those obtained from the differential gene analysis to create a new feature set for further screening.

**Figure 4:**
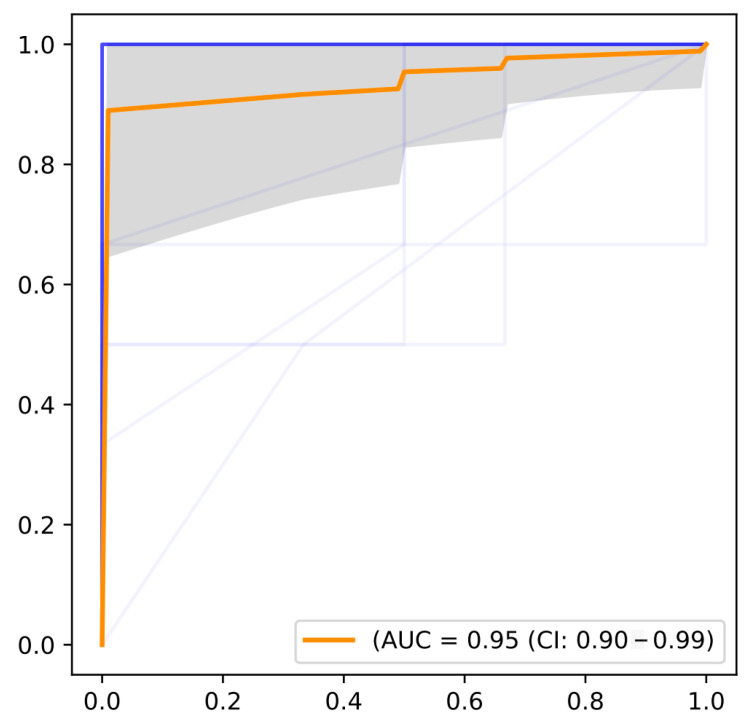
AUROC curves of the algorithms when applying RFE. The blue curve represents the ROC curve of a single cross-validation, while the orange curve indicates the average ROC curve. The gray area denotes the fluctuation range of the True Positive Rate.

To further reduce the number of genes, we applied RFE, and the AUROC curves for the results are shown in Figure 4. Ultimately, we screened out 151 genes, which were ranked by importance and prepared for further screening.

### 2.5. Further Screening of Genes

**Figure 5:**
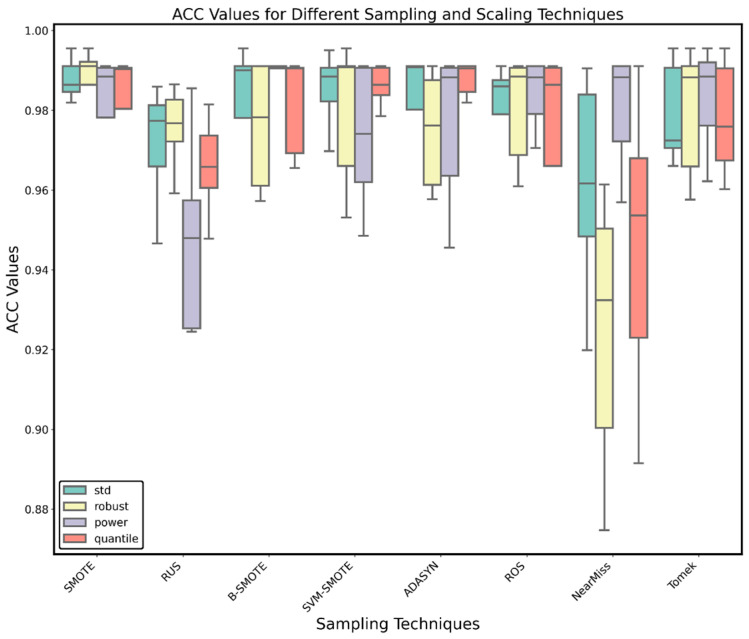
Box plots of ACC for the algorithms using different scaling methods and sampling techniques.

To further refine the selection of key genes, we incorporated data from the GEO database into the analysis, combined with the previously screened 151 genes, forming a new dataset. We constructed models and evaluated them by combining different sampling methods, scaling methods, and numbers of features. As shown in Figure 5, the best scaling method was standard scaling (std), with an average ACC of 97.75%, and the best sampling method was SMOTE, with an average ACC of 98.33%, both yielding good results.

**Figure 6:**
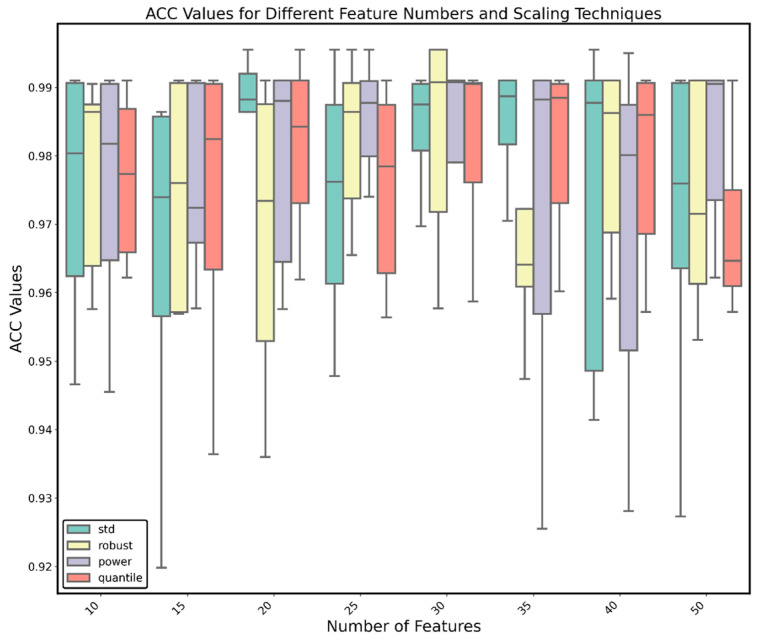
Box plots of ACC for the algorithms using different scaling methods and feature numbers.

To determine the number of selected features, we analyzed the impact of different feature numbers on the model results (Figure 6). The results showed that the best performance was achieved with 30 features, with an average ACC of 98.04%. Therefore, we ultimately selected the top 30 genes as important genes.

**Figure 7:**
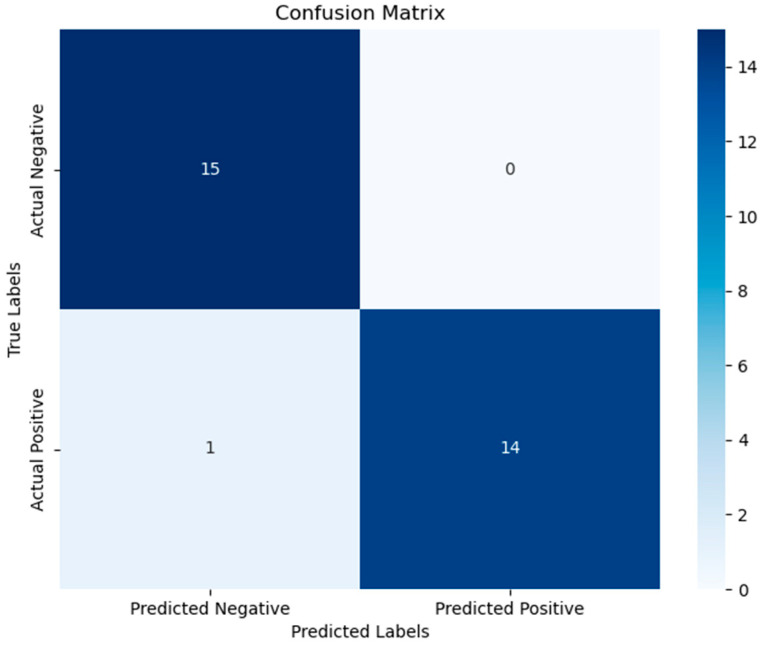
Confusion matrices of the best models obtained with different combinations of feature numbers, scaling methods, and sampling techniques.

Next, we evaluated the best model obtained from the above combinations, and its confusion matrix is shown in Figure 7, indicating good discrimination ability. Combining the above analysis with the results of the PPI and enrichment analyses, we found that genes such as ARC, EGR2, EGR3, FOS, FOSB, and JUNB appeared multiple times, suggesting they may be key genes in the process of VVTT infection in cells.

### 2.6. Quantitative Real-Time PCR (RT-qPCR) Analysis

**Figure 8:**
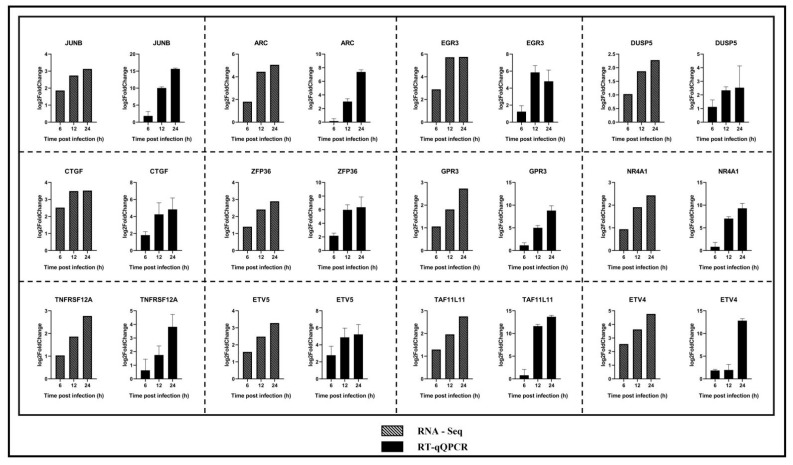
Matrices of the best models obtained with different combinations of feature numbers, scaling methods, and sampling techniques. Expression levels of genes JUNB, ARC, EGR3, DUSP5, CTGF, ZFP36, GPR3, NR4A1, TNFRSF12A, ETV5, TAF11L11, and ETV4 were validated by RT-qPCR. The NADPH gene was used as an internal control, and the relative quantity of gene expression (fold change) of each gene was calculated using the comparative 2^−ΔΔCt^ method. RT-qPCR values are shown as the mean ± SD.

To validate the reproducibility and repeatability of the DEGs identified from transcriptome sequencing, we selected twelve genes—JUNB, ARC, EGR3, DUSP5, CTGF, ZFP36, GPR3, NR4A1, TNFRSE12A, ETV5, TAF11L11, and ETV4—for RT-qPCR analysis (Figure 8). The results showed that these genes were significantly differentially expressed and consistently up- or down-regulated in line with the gene expression changes observed in RNA-Seq, indicating the reliability of the DEGs obtained from transcriptome sequencing.

The trends in differential expression of these genes confirmed by RT-qPCR in the mock vs. VVTT6 h, mock vs. VVTT12 h, and mock vs. VVTT24 h groups were consistent with those of the transcriptome sequencing results. This indicates that these genes are indeed up-regulated or down-regulated during infection and may play important roles in the infection process.

### 2.7. Discussion

In the field of *Orthopoxvirus* research, the integration of machine learning and transcriptomics has emerged as a potent tool. For instance, studies have utilized machine learning models to predict drugs against the monkeypox virus (MPXV) [41] or identified conserved surface sites of MPXV through immunoinformatics [42]. These studies offer valuable insights, particularly in enhancing the efficiency of vaccine and drug development through computational models. Poxvirus can exploit aberrant cell tumor suppression signals, such as the P53 signaling pathway, to enhance its oncolytic specificity and efficacy [43]. Our analysis confirms the engagement of pathways like spinocerebellar ataxia and the p53 signaling pathway during the infection process. These results were obtained by integrating high-throughput gene expression data with machine learning algorithms to identify potential key genes and signaling pathways during VVTT infection. The selection of these genes and pathways not only offers new insights into the pathogenic mechanisms of VVTT but also holds theoretical implications for future vaccine enhancements.

Since the eradication of smallpox, research on VVTT vaccines has been scarce, with a notable absence of human experimental data compared to other vaccine studies. Insufficient reliable data sources exist regarding the current analytical methods and results of VVTT, indicating numerous areas warranting further investigation. Particularly noteworthy are the recurring outbreaks of monkeypox, which, because of their close phylogenetic relationship, can serve as a breakthrough point if research data on monkeypox can be effectively consolidated and leveraged. Our current research primarily relies on computational simulations, leaving gaps in our understanding of the actual disease mechanisms. Further investigation is necessary to elucidate these mechanisms.

Furthermore, the experiment’s use of only three sub-holes per group may have resulted in a limited sample size for machine learning, potentially introducing more noise and requiring additional validation of the outcomes. Furthermore, while cross-validation was employed to minimize overfitting, the small sample size and high number of features could still pose a risk of overfitting, warranting further experimental scrutiny.

## 3. Materials and Methods

### 3.1. Datasets

Our dataset comprised transcriptome data obtained from our own experiments as well as data retrieved from the GEO database, specifically GSE36854 and GSE11238 [44]. To ensure the rigor of our data, we identified common gene expression data between our measured data and the GEO database entries and merged them into a new dataset. This newly formed dataset was subsequently used for machine learning in the following steps.

### 3.2. Virus and Cells

Human Embryonic Kidney 293A (A293) cells were maintained at 37 °C, 5% CO_2_ in a high-glucose Dulbecco’s modified Eagle’s medium (DMEM; Gibco, Waltham, MA, USA) that was supplemented with 10% fetal bovine serum (FBS; Gibco, Waltham, MA, USA), 10 mM 4-(2-hydroxyethyl)-1-piperazineethanesulfonic acid (HEPES; Gibco, Waltham, MA, USA), and 1% Penicillin Streptomycin (P/S; Gibco, Waltham, MA, USA). The 293A cells were purchased from the Cell Resource Center, Institute of Basic Medical Sciences (CAMS/PUMC), Beijing China, Laboratory Preservation and Subculture. The VVTT strain (GenBank accession no. AF095689) was obtained from the Institute of Virology at the Chinese Center for Disease Control and Prevention.

### 3.3. Sample Collection and RNA Extraction

The 293A cells were plated into a 6-well cell culture plate (Corning) in DMEM with 10% FBS until 5 × 105 cells/well density. The 293A cells were inoculated with 0.1 MOI VVTT and maintained at 37 °C, 5% CO_2_ for 1 h [45,46]. Afterward, the cells were washed thrice with PBS and grown in DMEM with 1% FBS. Samples were collected at 6, 12, and 24 h post-infection based on the growth curve pattern [47,48]. The experiment was performed with three biological replicates for error reduction.

### 3.4. Transcriptome Analysis

Total RNA was extracted using TRIzol^®^ Reagent (Invitrogen, Waltham, Massachusetts, USA) according to the manufacturer’s instructions. Subsequently, samples were collected for transcriptome analysis. Quality control was performed on the raw data to remove low-quality data and noise. The sequencing data were then mapped to the reference genome, followed by normalization to determine gene expression levels. Finally, the differences in gene expression among different samples were analyzed.

We conducted differential gene analysis by comparing samples infected with VVTT at 6, 12, and 24 h to uninfected A293 cells at corresponding time points. The criteria for differential gene selection were a fold change greater than 2 and a *p*-value less than 0.05. The identified differentially expressed genes were then imported into STRING https://cn.string-db.org/ (accessed on 21 June 2024) and visualized using Cytoscape (Version: 3.10.1) [49,50]. The top 20 genes were selected as key genes based on their degree of centrality.

Next, we performed Gene Ontology (GO) and Kyoto Encyclopedia of Genes and Genomes (KEGG) pathway analyses on the differentially expressed genes [51,52,53]. Similarly, the top 20 pathways were selected as key pathways for further analysis.

### 3.5. Preliminary Screening

We employed algorithms such as random forest (RF), lasso regression (LASSOCV), elastic net (EN), and eXtreme Gradient Boosting (XGB) for feature selection, using the previously obtained expression matrix as the dataset. For RF, we used ExtraTreesClassifier with 500 estimators and a random state of 42 to ensure reproducibility. For LASSOCV, we utilized LassoCV with a random state of 42 and 50 parallel jobs. For EN, we employed ElasticNetCV with 5-fold cross-validation, a maximum of 10,000 iterations, a range of alpha values from 0.001 to 10, and l1_ratio values from 0.1 to 0.9. For XGB, we used XGBClassifier with 300 estimators, a learning rate of 1.0, a maximum depth of 3, and a random state of 42.

Five-fold cross-validation was performed and repeated twice. The features selected by these four algorithms were combined with those identified through differential gene analysis for the next round of screening. Additionally, accuracy (ACC), the area under the curve (AUC), Matthews correlation coefficient (MCC), sensitivity (SEN), and specificity (SPE) were used to evaluate each algorithm, and the best-performing algorithm was chosen for further analysis.

Next, we applied Recursive Feature Elimination (RFE) to further refine the selected features. The BRF function utilizes the following parameters: class_weight = “balanced_subsample” addresses class imbalance by assigning balanced weights to each class during training. Hyperparameter tuning is performed using param_grid to adjust three key parameters: n_estimators (100, 200, 300, 400, 500) controls the forest size; max_features (“sqrt: and “log2:) determines the number of features for tree splitting; max_depth (1, 3, 5, 7, 9) limits tree depth to prevent overfitting or underfitting. Furthermore, cv = 3 employs 3-fold cross-validation for model evaluation to ensure generalization ability. These parameters, combined with feature selection and hyperparameter optimization, train an optimized random forest classifier. The area under the receiver operating characteristic curve (AUROC) was generated for the analysis, and the genes were ranked by their importance from highest to lowest, preparing them for the next step of analysis.

### 3.6. Further Screening

We combined the selected data from the GEO database with the expression matrix of differentially expressed genes, using the genes and their expression levels identified in the previous step to form a new dataset. Using XGB as the base algorithm, we combined different scaling methods (StandardScaler, QuantileTransformer, PowerTransformer, and RobustScaler) and different sampling methods (Synthetic Minority Over-sampling Technique (SMOTE), Adaptive Synthetic Sampling Approach (ADASYN), Random Under-sampling (RUS), Random Over-sampling (ROS), Borderline-SMOTE (B-SMOTE), Support Vector Machine-SMOTE (SVM-SMOTE), Tomek Links, and NearMiss) and numbers of features, performing five-fold cross-validation and repeating twice. The performance of the algorithms was evaluated with the ACC score. The best algorithm was selected, and a confusion matrix was plotted. The selected features were comprehensively evaluated and combined with PPI and pathway enrichment analyses to identify the key features.

### 3.7. Validation of Transcriptome Sequencing Results

Reverse transcription quantitative real-time PCR (RT-qPCR) was performed to validate the DEGs identified from transcriptome sequencing. Twelve DEGs, namely, JUNB, ARC, EGR3, DUSP5, CTGF, ZFP36, GPR3, NR4A1, TNFRSE12A, ETV5, TAF11L11, and ETV4, were selected for RT-qPCR validation and analyses of gene expression. Total RNA extraction was performed using TRIzol reagent (Invitrogen, Waltham, Massachusetts, USA) following the manufacturer’s instructions. Sequence-specific primers of genes were designed using the NCBI website design (Table 3). Fastking One Step Probe RT-qPCR (Probe) was used to perform RT-qPCR following the manufacturer’s instructions. RT-qPCR was performed in 20 μL reaction volumes containing 10 μL of 2× Fastking One Step Probe RT-qPCR MasterMix, 0.8 μL of 2× Fastking Enzyme Mix, 0.5 μL of upstream and downstream primers (10 μM), 0.4 μL of probe (10 μM), 1 μL of RNA template, and 6.8 μL of ddH_2_O. The following reaction profile was used: 50 °C for 30 min, 95 °C for 3 min, followed by 40 cycles of 95 °C for 15 s and 60 °C for 30 s; melting curve analysis was performed to validate specific amplification. The NADPH gene was used as an endogenous reference gene. RT-qPCR was performed in a 96-well plate on a BIO RAD CFX96 Real-Time System (Bio-Rad CFX Maestro, Singapore). The detection was performed in triplicate for each biological replicate. The relative expression values of selected genes were calculated using the 2^−ΔΔCt^ method and normalized against the expression levels of the NADPH gene.

## 4. Conclusions

Through enrichment analysis, we found that pathways such as amphetamine addiction, circadian entrainment, MAPK signaling pathway, osteoclast differentiation, C-type lectin receptor signaling pathway, and TNF signaling pathway are closely related to VVTT infection in A293 cells, explaining some of the changes that occur during VVTT infection.

By combining transcriptome analysis and machine learning, we identified four key targets related to VVTT infection in A293 cells: ARC, JUNB, EGR3, and FOS.

Finally, through qPCR analysis, we validated the up-regulation of these four targets following VVTT infection, indicating their potential importance in the VVTT infection process. Taken together, our integrated approach using transcriptomics and machine learning has provided a deeper understanding of the potential mechanisms underlying VVTT infection in A293 cells and identified potential key regulatory targets. Research on these targets not only offers new insights into the molecular mechanisms of VVTT infection but also provides a scientific basis and direction for developing future vaccines and therapeutic strategies against VVTT. We have included plans for further validation of target genes through gene-targeted knockdown and overexpression techniques. Subsequently, we aim to compare genetic alterations at the cellular level post-infection of A293 cells with the VVTT-modified vaccine strain, known for its reduced toxicity and enhanced safety, alongside the VVT vaccine strain. Furthermore, we will conduct efficacy validation experiments on known targeted drugs for the genes obtained in the study. This study lays a solid foundation for subsequent in-depth research on VVTT infection and related diseases and offers potential targets and ideas for developing new antiviral drugs.

## Figures and Tables

**Table 1:** Top 20 genes ranked by degree in the PPI network.

RANK	VVTT_6 h vs. A293_6 h	VVTT_12 h vs. A293_12 h	VVTT_24 h vs. A293_24 h
1	FOS	FOS	FOS
2	EGR2	EGR1	EGR1
3	EGR1	FOSB	ATF3
4	DUSP1	NR4A1	FOSB
5	FOSB	JUNB	NR4A1
6	ZFP36	IER2	JUNB
7	JUNB	EGR2	EGR2
8	IER2	ZFP36	DUSP1
9	EGR3	DUSP1	ZFP36
10	H3C13	EGR3	IL6
11	H3C12	DUSP2	IER2
12	PTGS2	GADD45B	EGR3
13	IER3	FOSL1	FOSL2
14	H4C6	ARC	FOSL1
15	H2AC8	H3C13	GADD45B
16	ARC	H3C12	PTGS2
17	SOCS3	CCN1	DUSP2
18	DUSP6	H4C6	CSRNP1
19	CCN2	H2AC8	DUSP5
20	BHLHE40	H2AC20	CCN1

**Table 2:** Top 20 pathways ranked in the KEGG analysis.

Rank	A6 h_VS._M6 h	A12 h_VS._M12 h	A24 h_VS._M24 h
1	HIF-1 signaling	Carbon metabolism	Hippo signaling
2	Cell cycle	Cellular senescence	Cocaine addiction
3	Longevity regulating	Biosynthesis of amino acids	p53 signaling
4	Autophagy-animal	HIF-1 signaling pathway	Insulin resistance
5	Human papillomavirus infection	Huntington disease	Arginine and proline metabolism
6	Efferocytosis	Protein processing in endoplasmic reticulum	MAPK signaling pathway
7	p53 signaling pathway	Cell cycle	Autophagy-animal
8	Proteasome	Endocytosis	Amphetamine addiction
9	Protein processing in endoplasmic reticulum	FoxO signaling pathway	Protein processing in endoplasmic reticulum
10	Proteoglycans in cancer	Autophagy-animal	Hepatocellular carcinoma
11	FoxO signaling pathway	Bladder cancer	AMPK signaling pathway
12	TNF signaling pathway	AMPK signaling pathway	Insulin signaling pathway
13	Chronic myeloid leukemia	Human T-cell leukemia virus 1 infection	Breast cancer
14	Spinocerebellar ataxia	Central carbon metabolism in cancer	Cell cycle
15	mTOR signaling pathway	mTOR signaling pathway	Spinocerebellar ataxia
16	Thyroid hormone signaling pathway	Spinocerebellar ataxia	Synaptic vesicle cycle
17	Insulin resistance	Non-small cell lung cancer	Notch signaling pathway
18	Biosynthesis of amino acids	Hepatocellular carcinoma	Lysosome
19	DNA replication	Hepatitis B	Longevity regulating pathway
20	Hepatocellular carcinoma	p53 signaling pathway	Bladder cancer

**Table 3:** Primer name and sequence

Gene Name	Prime Sequence (5′-3′)	Gene Name	Prime Sequence (5′-3′)	Gene Name	Prime Sequence (5′-3′)
GAPDH-F	TCAAGCTCATTTCCTGGTATGACA	GAPDH-R	GGGTCTTACTCCTTGGAGGC	GAPDH-P	TGGTGGACCTCATGGCCCACA
JUNB-F	CGACCACCATCAGCTACCTC	JUNB-R	GTCTGCGGTTCCTCCTTGAA	JUNB-P	CTTCGCCGGTGGCCACCC
ARC-F	GGCCCCTCAGCTCCAGT	ARC-R	GACAGCTGATGGTGGGGTC	ARC-P	GGCAGCAGCTGGCACCATCA
EGR3-F	CGCGGTGGGAGAGAGAATG	EGR3-R	GTTGGAAGGGGAGTCGAAGG	EGR3-P	CCCCCGGCAACAAGACCGTG
DUSP5-F	AGCTTATGACCAGGGTGGC	DUSP5-R	GTCGGGAGACATTCAGCAGG	DUSP5-P	CGAGTTCCTCGCCAACCTGCA
CTGF-F	GGAGTGGGTGTGTGACGAG	CTGF-R	CTTCCAGTCGGTAAGCCGC	CTGF-P	AAACCGTGGTTGGGCCTGCC
ZFP36-F	TGACTGCCATCTACGAGAGCC	ZFP36-R	GTCCCTCCATGGTCGGATGG	ZFP36-P	GTCGCTGAGCCCTGACGTGC
GPR3-F	GTGACTCACGCCGCTTCT	GPR3-R	CCACATCATGGTACCGCTCA	GPR3-P	GCTTCTCGGGGTCCACGCAC
NR4A1-F	GACCCCGGAAAGCGGG	NR4A1-R	TGGATACAGGGCATCTCACTCTG	NR4A1-P	GGCAGCCTGGCTCCTTCTGC
TNFRSF12A-F	AGAGAGAAGTTCACCACCCC	TNFRSF12A-R	GCACATTGTCACTGGATCAGC	TNFRSF12A-P	GAGGAGACCGGCGGAGAGGG
ETV5-F	AACCGGAAGAGGTTGCTCG	ETV5-R	GGCATCTGGGTCACAGACAA	ETV5-P	GCGCTGGGGCATCCAGAAGA
TAF11L11-F	TGGCACAGCAGAAACACAGAA	TAF11L11-R	TGAGATTCCAGTCTGCTCCG	TAF11L11-P	TCGAACCACCCCAGCCAGAACT
ETV4-F	CGCCTACGACTCAGATGTCA	ETV4-R	GGTTTCTCATAGCCATAGCCCA	ETV4-P	CTCTCCAGGTGACGGGGCCA

## Data Availability

Data will be provided upon request.

## Supplementary Materials

The following supporting information can be downloaded at https://www.mdpi.com/article/10.3390/ijms26031203/s1.

## Abbreviations

The following abbreviations are used in this manuscript:

VV	Vaccinia virus
VVTT	Vaccinia virus Tiantan
GO	Gene Ontology
KEGG	Kyoto Encyclopedia of Genes and Genomes
RF	Random forest
LASSOCV	Least absolute shrinkage and selection operator
EN	Elastic net
XGB	eXtreme Gradient Boosting
RFE	Recursive Feature Elimination
ACC	Accuracy
AUROC	Area under the receiver operating characteristic curve
MCC	Matthews correlation coefficient
Sen	Sensitivity
Spe	Specificity
AUC	Area under the curve
DEG	Differentially expressed genes
PPI	Protein–protein interaction
