# Correction: Chen et al. Elucidating the Mechanism of VVTT Infection Through Machine Learning and Transcriptome Analysis. *Int. J. Mol. Sci.* 2025, *26*, 1203

**DOI:** 10.3390/ijms27073330

**Published:** 2026-04-07

**Authors:** Zhili Chen, Yongxin Jiang, Jiazhen Cui, Wannan Li, Weiwei Han, Gang Liu

**Affiliations:** 1 Academy of Military Medical Sciences, Beijing 100850, China; chenzl0708@163.com (Z.C.); cjiazhen2023@163.com (J.C.); 2 Key Laboratory for Molecular Enzymology and Engineering of Ministry of Education, School of Life Sciences, Jilin University, 2699 Qianjin Street, Changchun 130012, China; jiangyx23@mails.jlu.edu.cn (Y.J.); weiweihan@jlu.edu.cn (W.H.)

In the original publication [1], there was a mistake in Figure 8 as published. The authors mistook the 2^−ΔΔCt^ value for the Lg 2FC value, resulting in a numerical calculation error in Figure 8. The corrected Figure 8 appears below. The authors state that the scientific conclusions are unaffected. This correction was approved by the Academic Editor. The original publication has also been updated.

## Figures and Tables

**Figure 8:**
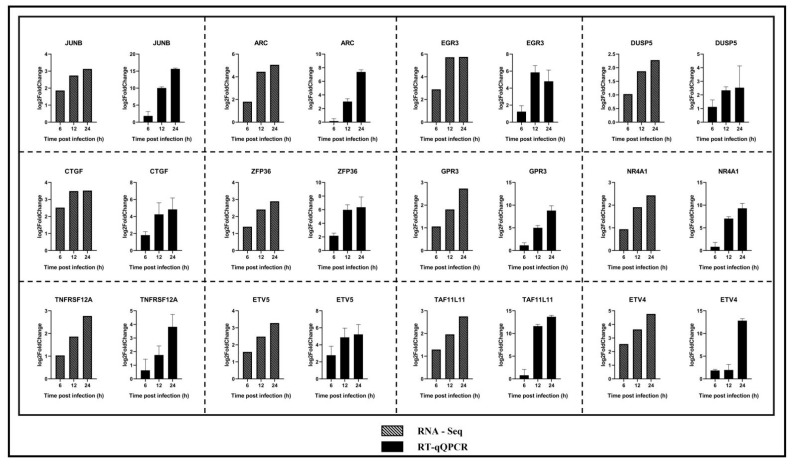
Matrices of the best models obtained with different combinations of feature numbers, scaling methods, and sampling techniques. Expression levels of genes JUNB, ARC, EGR3, DUSP5, CTGF, ZFP36, GPR3, NR4A1, TNFRSF12A, ETV5, TAF11L11, and ETV4 were validated by RT-qPCR. The NADPH gene was used as an internal control, and the relative quantity of gene expression (fold change) of each gene was calculated using the comparative 2^−ΔΔCt^ method. RT-qPCR values are shown as the mean ± SD.
