# Supplementary material for: Elucidating the Mechanism of VVTT Infection Through Machine Learning and Transcriptome Analysis
Source: Int J Mol Sci. 2025 Jan 30;26(3):1203. doi: 10.3390/ijms26031203 (PMC11818747; doi:10.3390/ijms26031203)
Supplement: Supplementary file 1 [file ijms-26-01203-s001.zip › ijms-3405357-supplementary.pdf]

Supplemental Materials

Figure S1. The GO analysis results between Mock and VVTT6h, sorted by Padjust.
Figure S2. The GO analysis results between Mock and VVTT12h, sorted by Padjust.
Figure S3. The GO analysis results between Mock and VVTT24h, sorted by Padjust.
Figure S4. The KEGG analysis results between Mock and VVTT6h, sorted by Padjust.
Figure S5. The KEGG analysis results between Mock and VVTT12h, sorted by Padjust.
Figure S6. The KEGG analysis results between Mock and VVTT24h, sorted by Padjust.

Figure S1

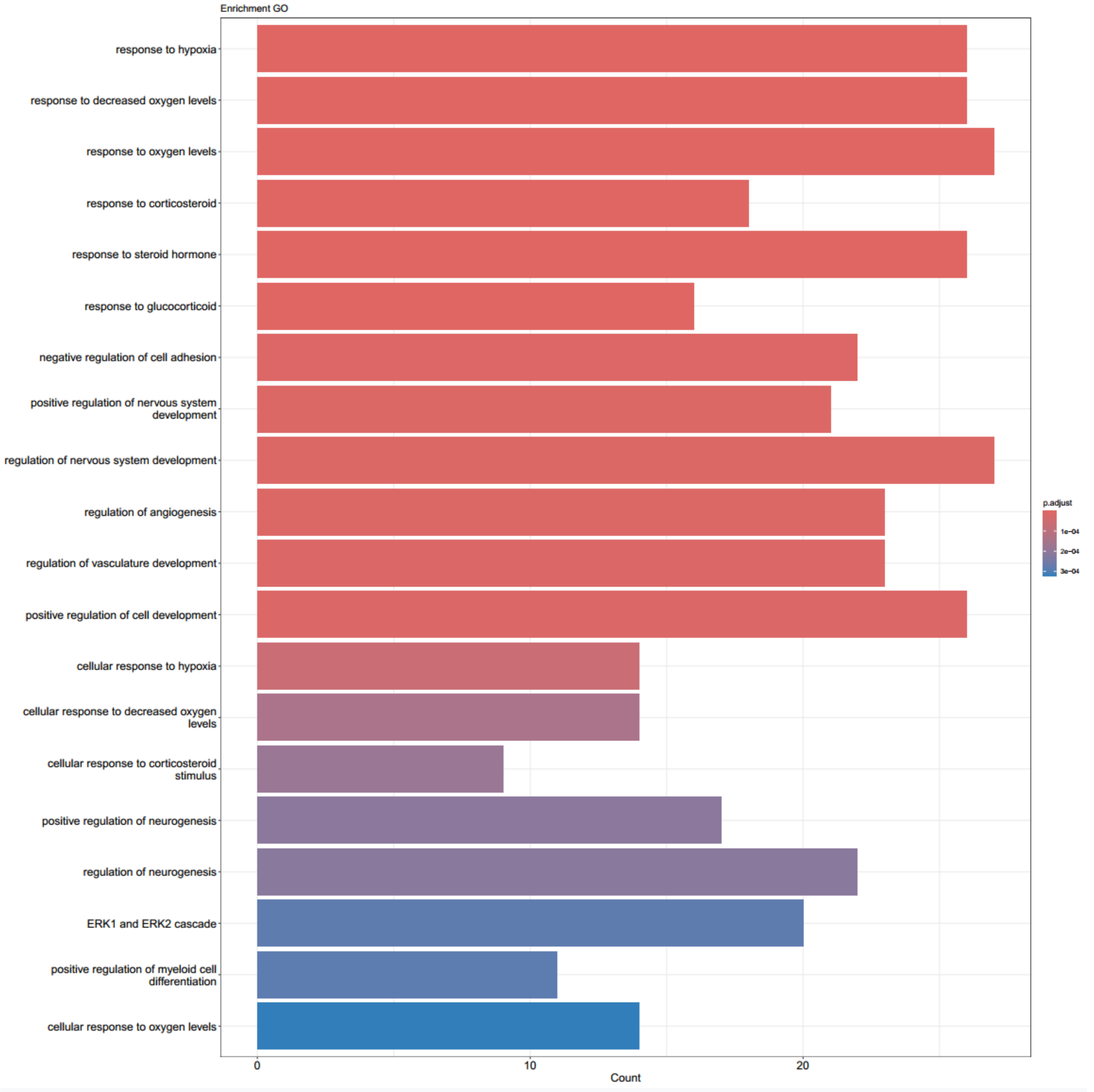

Enrichment GO
response to hypoxia
response to decreased oxygen levels
response to oxygen levels
response to corticosteroid
response to steroid hormone
response to glucocorticoid
negative regulation of cell adhesion
positive regulation of nervous system development
regulation of nervous system development
regulation of angiogenesis
regulation of vasculature development
positive regulation of cell development
cellular response to hypoxia
cellular response to decreased oxygen levels
cellular response to corticosteroid stimulus
positive regulation of neurogenesis
regulation of neurogenesis
ERK1 and ERK2 cascade
positive regulation of myeloid cell differentiation
cellular response to oxygen levels
0
10
20
Count
p.adjust
1e-04
2e-04
3e-04

Figure S2

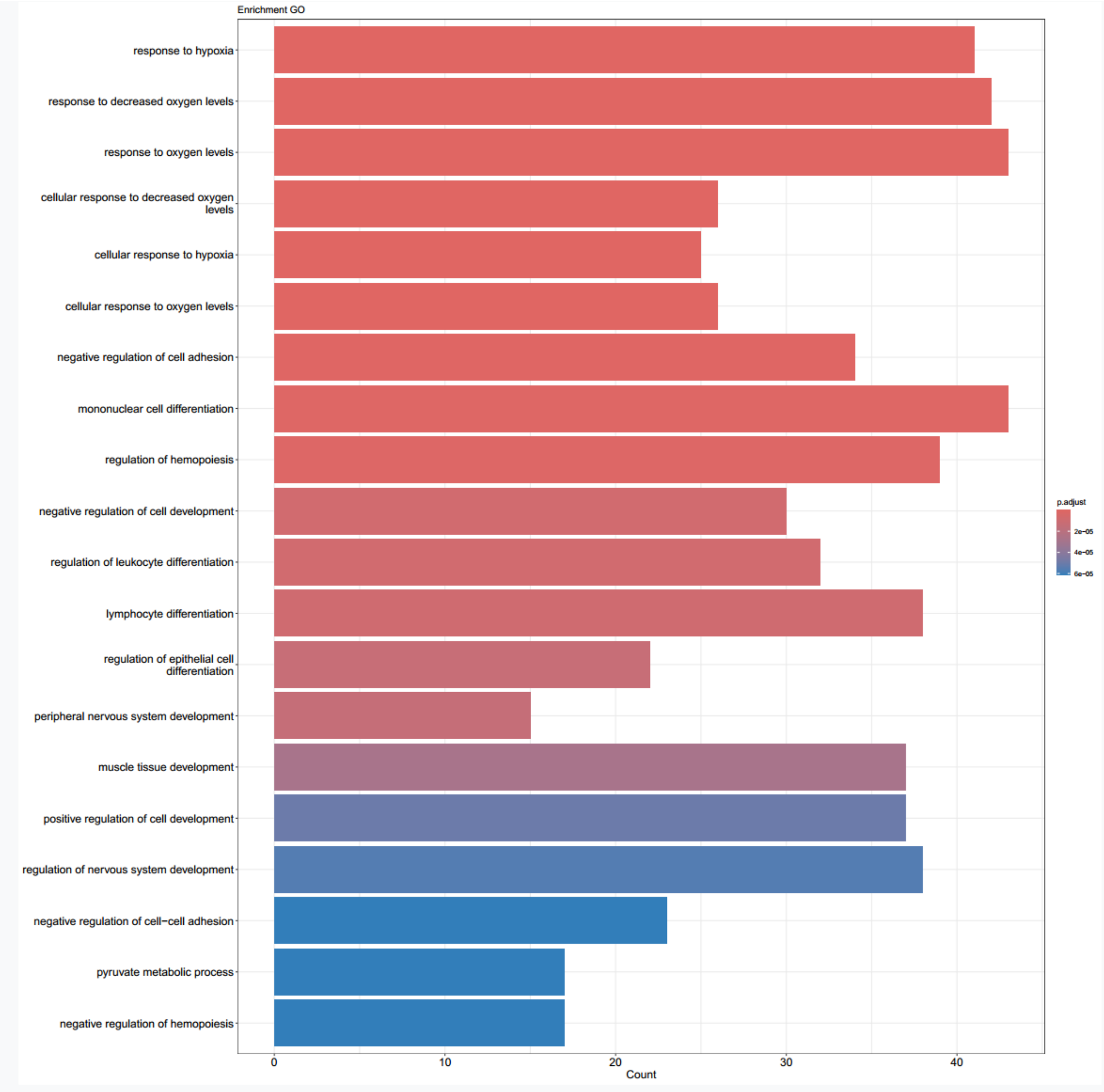
Enrichment GO
response to hypoxia
response to decreased oxygen levels
response to oxygen levels
cellular response to decreased oxygen levels
cellular response to hypoxia
cellular response to oxygen levels
negative regulation of cell adhesion
mononuclear cell differentiation
regulation of hemopoiesis
negative regulation of cell development
regulation of leukocyte differentiation
lymphocyte differentiation
regulation of epithelial cell differentiation
peripheral nervous system development
muscle tissue development
positive regulation of cell development
regulation of nervous system development
negative regulation of cell−cell adhesion
pyruvate metabolic process
negative regulation of hemopoiesis
0
10
20
30
40
Count
p.adjust
2e−05
4e−05
6e−05

Figure S3

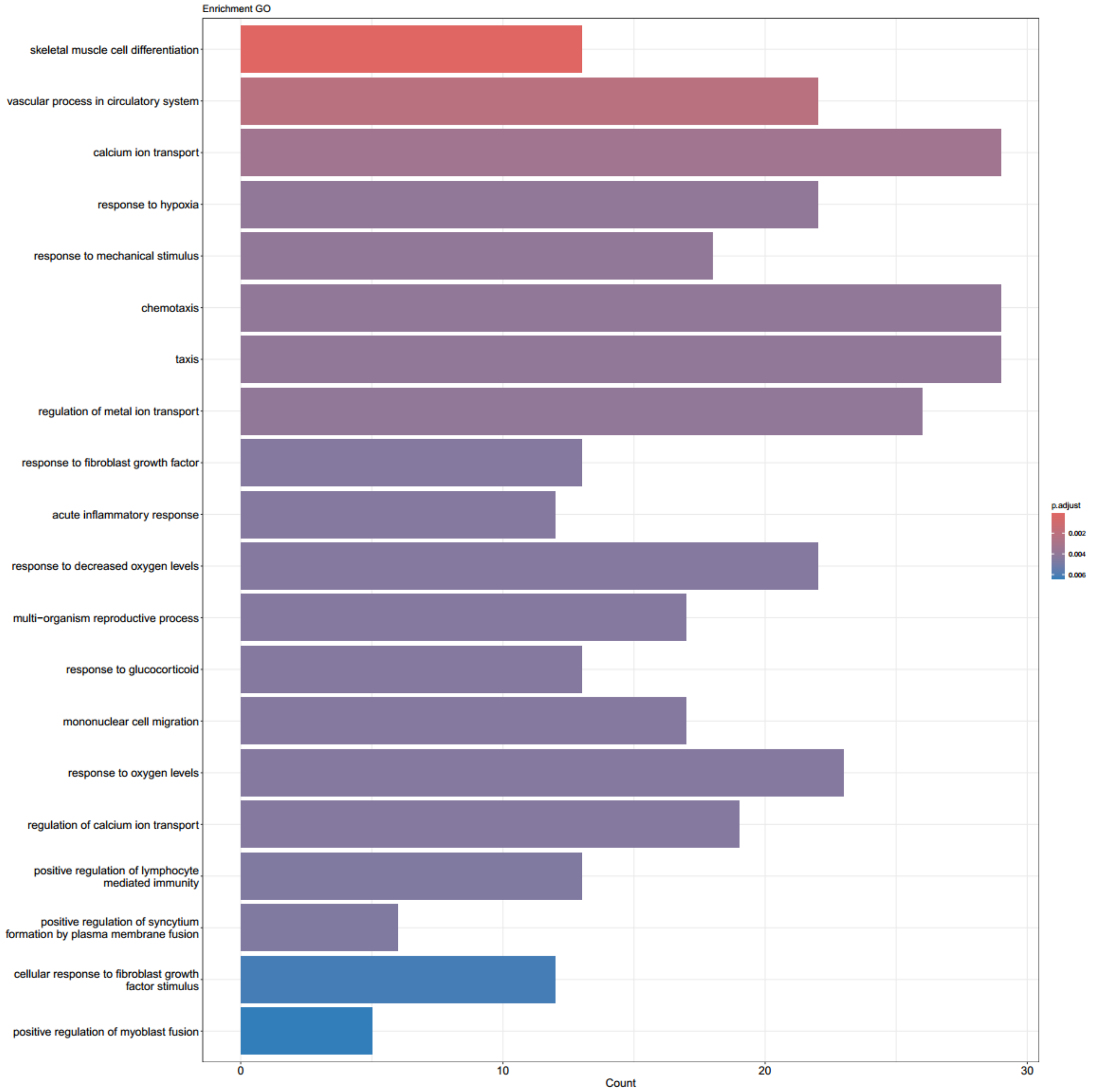
Enrichment GO
skeletal muscle cell differentiation
vascular process in circulatory system
calcium ion transport
response to hypoxia
response to mechanical stimulus
chemotaxis
taxis
regulation of metal ion transport
response to fibroblast growth factor
acute inflammatory response
response to decreased oxygen levels
multi−organism reproductive process
response to glucocorticoid
mononuclear cell migration
response to oxygen levels
regulation of calcium ion transport
positive regulation of lymphocyte mediated immunity
positive regulation of syncytium formation by plasma membrane fusion
cellular response to fibroblast growth factor stimulus
positive regulation of myoblast fusion
0
10
20
30
Count
p.adjust
0.002
0.004
0.006

Figure S4

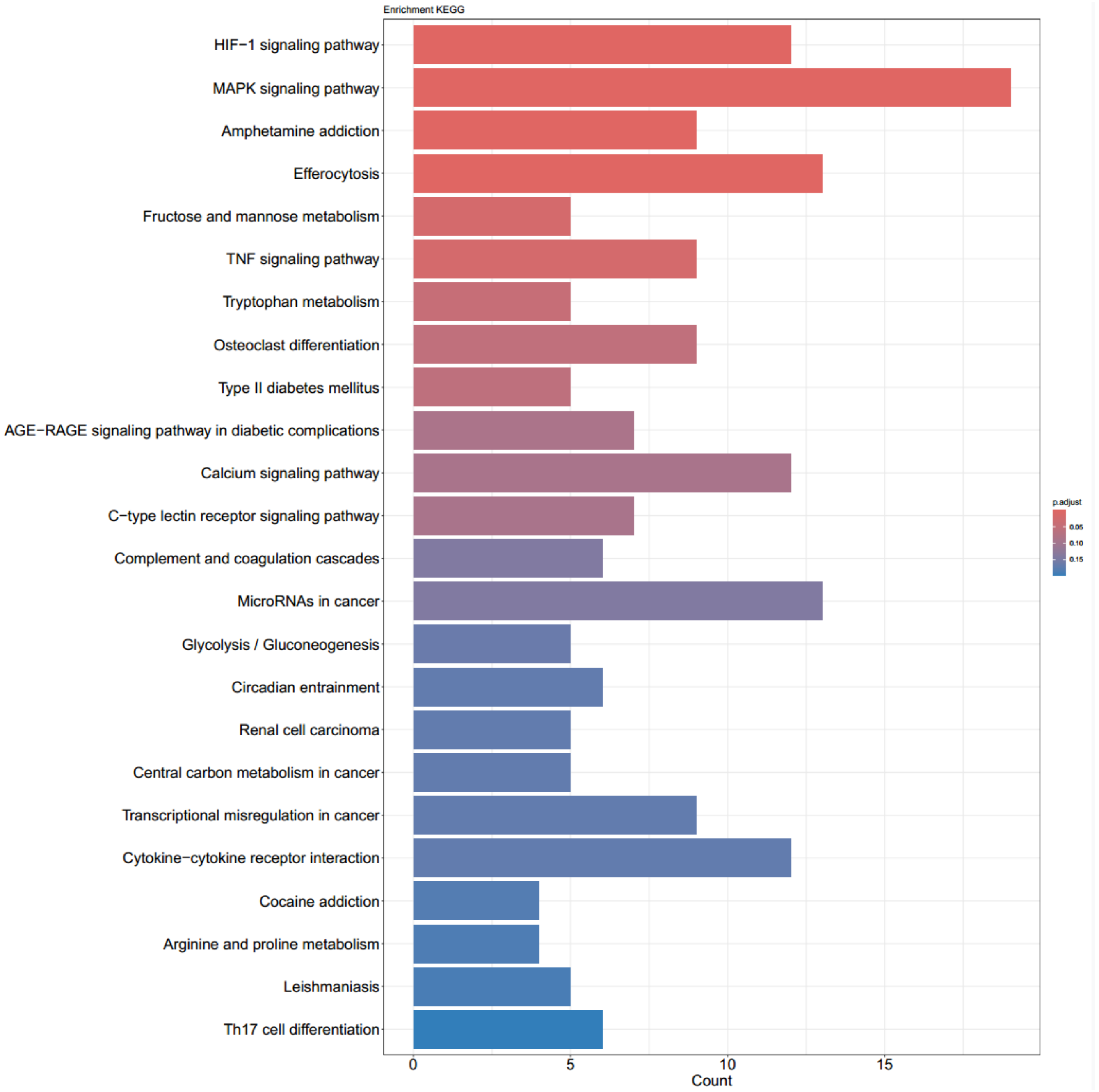

Enrichment KEGG
HIF−1 signaling pathway
MAPK signaling pathway
Amphetamine addiction
Efferocytosis
Fructose and mannose metabolism
TNF signaling pathway
Tryptophan metabolism
Osteoclast differentiation
Type II diabetes mellitus
AGE−RAGE signaling pathway in diabetic complications
Calcium signaling pathway
C−type lectin receptor signaling pathway
Complement and coagulation cascades
MicroRNAs in cancer
Glycolysis / Gluconeogenesis
Circadian entrainment
Renal cell carcinoma
Central carbon metabolism in cancer
Transcriptional misregulation in cancer
Cytokine−cytokine receptor interaction
Cocaine addiction
Arginine and proline metabolism
Leishmaniasis
Th17 cell differentiation
0
5
10
15
Count
p.adjust
0.05
0.10
0.15

Figure S5

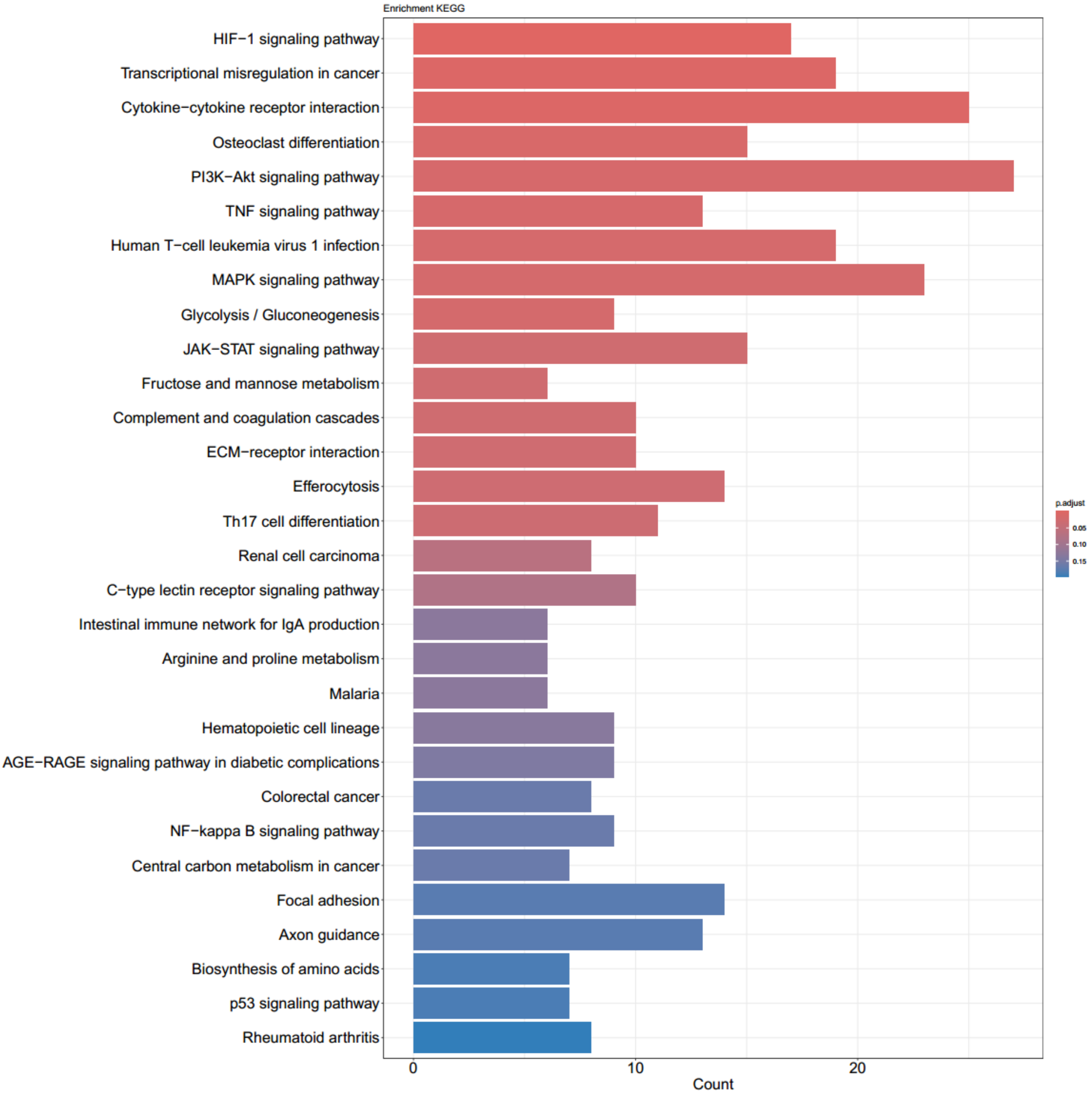

Enrichment KEGG
HIF−1 signaling pathway
Transcriptional misregulation in cancer
Cytokine−cytokine receptor interaction
Osteoclast differentiation
PI3K−Akt signaling pathway
TNF signaling pathway
Human T−cell leukemia virus 1 infection
MAPK signaling pathway
Glycolysis / Gluconeogenesis
JAK−STAT signaling pathway
Fructose and mannose metabolism
Complement and coagulation cascades
ECM−receptor interaction
Efferocytosis
Th17 cell differentiation
Renal cell carcinoma
C−type lectin receptor signaling pathway
Intestinal immune network for IgA production
Arginine and proline metabolism
Malaria
Hematopoietic cell lineage
AGE−RAGE signaling pathway in diabetic complications
Colorectal cancer
NF−kappa B signaling pathway
Central carbon metabolism in cancer
Focal adhesion
Axon guidance
Biosynthesis of amino acids
p53 signaling pathway
Rheumatoid arthritis
0
10
20
Count
p.adjust
0.05
0.10
0.15

Figure S6

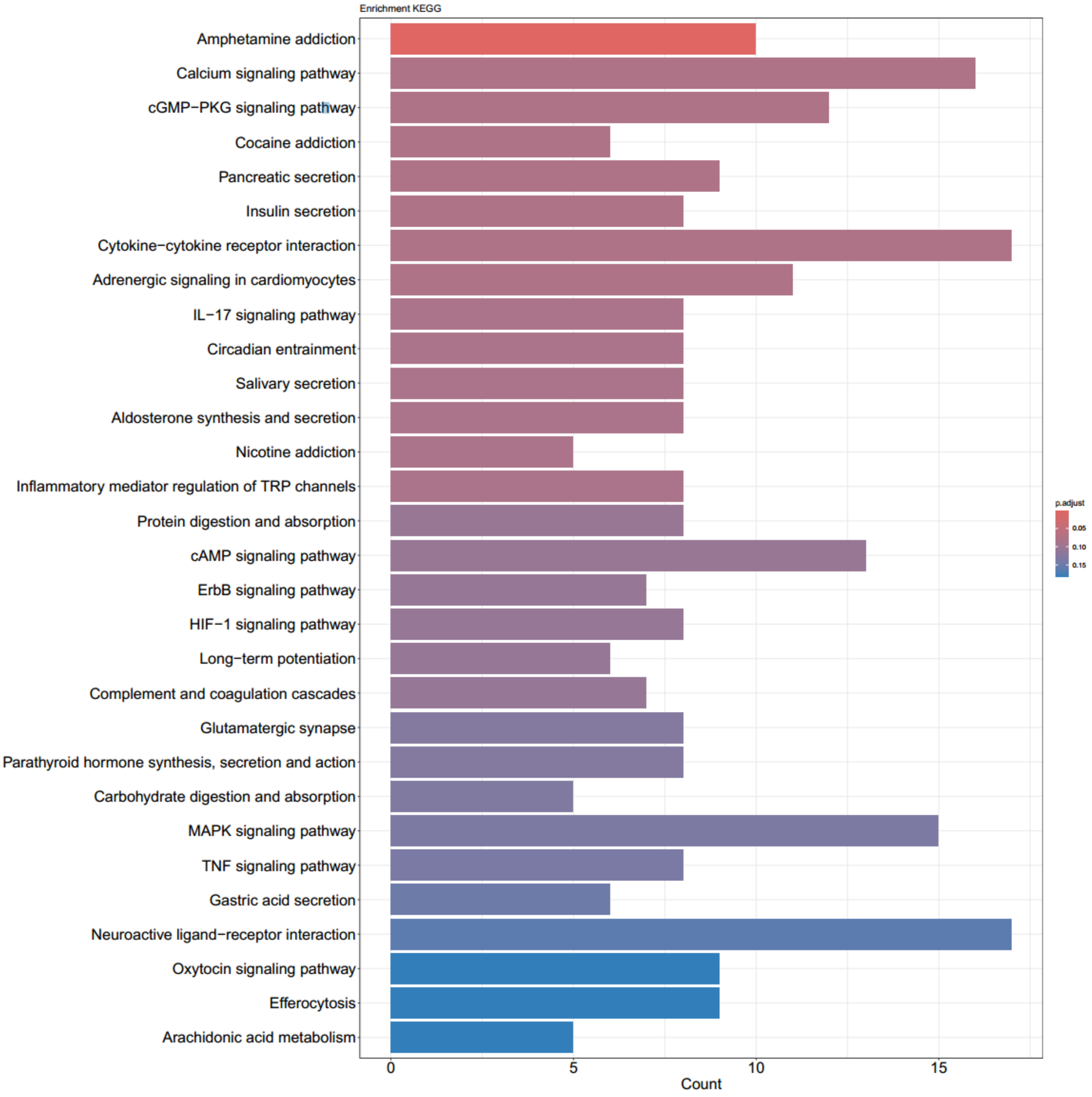

Enrichment KEGG
Amphetamine addiction
Calcium signaling pathway
cGMP−PKG signaling pathway
Cocaine addiction
Pancreatic secretion
Insulin secretion
Cytokine−cytokine receptor interaction
Adrenergic signaling in cardiomyocytes
IL−17 signaling pathway
Circadian entrainment
Salivary secretion
Aldosterone synthesis and secretion
Nicotine addiction
Inflammatory mediator regulation of TRP channels
Protein digestion and absorption
cAMP signaling pathway
ErbB signaling pathway
HIF−1 signaling pathway
Long−term potentiation
Complement and coagulation cascades
Glutamatergic synapse
Parathyroid hormone synthesis, secretion and action
Carbohydrate digestion and absorption
MAPK signaling pathway
TNF signaling pathway
Gastric acid secretion
Neuroactive ligand−receptor interaction
Oxytocin signaling pathway
Efferocytosis
Arachidonic acid metabolism
0
5
10
15
Count
p.adjust
0.05
0.10
0.15
